# Role of functionally dominant species in varying environmental regimes: evidence for the performance-enhancing effect of biodiversity

**DOI:** 10.1186/1472-6785-12-14

**Published:** 2012-07-30

**Authors:** Silke Langenheder, James I Prosser, Martin Solan

**Affiliations:** 1 Institute of Biological and Environmental Sciences, University of Aberdeen, Cruickshank Building, St. Machar Drive, Aberdeen, AB24 UU3, UK; 2 Oceanlab, University of Aberdeen, Main Street, Newburgh, Aberdeenshire, AB41 6AA, UK; 3 Department of Ecology and Genetics/Limnology, Uppsala University, Norbyvägen 18D, Uppsala, 75236, Sweden; 4 Present address: Ocean and Earth Science, National Oceanography Centre, Southampton, University of Southampton, Waterfront Campus, European Way, Southampton, SO14 3ZH, UK; 5 Present address: Biological Sciences, University of Derby, Keldleston Road, Derby, DE22 1GB, UK

**Keywords:** Insurance effect, Buffering effect, Resistance, Resilience, Environmental variability

## Abstract

**Background:**

Theory suggests that biodiversity can act as a buffer against disturbances and environmental variability via two major mechanisms: Firstly, a stabilising effect by decreasing the temporal variance in ecosystem functioning due to compensatory processes; and secondly, a performance enhancing effect by raising the level of community response through the selection of better performing species. Empirical evidence for the stabilizing effect of biodiversity is readily available, whereas experimental confirmation of the performance-enhancing effect of biodiversity is sparse.

**Results:**

Here, we test the effect of different environmental regimes (constant versus fluctuating temperature) on bacterial biodiversity-ecosystem functioning relations. We show that positive effects of species richness on ecosystem functioning are enhanced by stronger temperature fluctuations due to the increased performance of individual species.

**Conclusions:**

Our results provide evidence for the performance enhancing effect and suggest that selection towards functionally dominant species is likely to benefit the maintenance of ecosystem functioning under more variable conditions.

## Background

 It is anticipated that climate change impacts and subsequent environmental alterations, in combination with the effects of anthropogenic stressors, will have dramatic consequences for biodiversity, community composition and the level of ecosystem functioning [1,2]. It has been argued that biodiversity can counteract negative effects of environmental variability and disturbances (e.g. [3,4]) providing that sufficient species (or functionally important traits) can persist and continue to perform at opposite extremes of the fluctuating spectrum. Accordingly, it is hypothesized that species richness has an insurance effect and can maintain or increase ecosystem functioning via two major mechanisms [4]. The first involves a buffering effect, in which increasing species richness decreases the temporal variance of an ecosystem function. The second proposes a performance-enhancing effect through which higher species richness increases the mean of an ecosystem function over time. The buffering effect is driven by asynchrony of the species’ responses to environmental fluctuations [5], leading to more stable performance due to compensatory processes [6,7], whilst the performance-enhancing effect requires selection of the best-performing species, so that environmental fluctuations cause an increase in ecosystem functioning by strengthening the dominance of those particular species [4].

 Despite general acceptance that both of these biodiversity effects are likely to occur in natural systems, empirical studies that seek to test these mechanisms explicitly provide mixed results and have largely focused upon buffering effects at the expense of performance-enhancing effects. Several experimental studies have addressed the buffering effect of biodiversity on temporal variance in ecosystem functioning in systems with multiple pulse disturbances (i.e. fluctuating environments), only to find stabilizing, destabilizing or neutral effects of increasing diversity on stability (reviewed by [8]). Other studies have investigated the effect of biodiversity on ecosystem resistance and resilience in response to single disturbances [9]. Results from these studies have also not always supported the insurance hypothesis: Pfisterer and Schmid [10], for example, found that species richness decreased both resistance and resilience, in direct contradiction to theoretical predictions. Others have found positive effects of biodiversity on resilience, but not on resistance (e.g. [11,12]), or on resistance alone [13,14]. In contrast, explicit experimental demonstrations of the performance-enhancing effect are sparse [15], and most studies provide no, or only limited, support for a positive effect of diversity on the mean or magnitude of an ecosystem function under fluctuating or perturbed conditions [10,16-19].

Here we present a combinatory experiment to test the effect of temperature fluctuations on bacterial biodiversity-ecosystem functioning relationships over multiple generations. In this study we take advantage of the suitability that bacteria offer as a model system [20] and use bacterial communities consisting of 5 strains to examine theoretical predications of how environmental fluctuations affect the performance of multi-species communities. The 5 bacterial strains were assembled in all possible combinations and respiratory activities of communities in medium supplied with a single carbon source (glucose) was used as a proxy for ‘ecosystem functioning’. Communities were incubated for two days under five temperature fluctuation regimes, which varied with regard to the frequency and magnitude of the temperature change. We hypothesize that positive relationships between species richness and respiratory activities will be more pronounced when communities are exposed to temperature changes, i.e. species richness will have a performance-enhancing effect on ecosystem functioning under fluctuating conditions.

## Methods

Communities were constructed from combinations of five bacterial strains (**A**: *Flavobacterium* sp. SL-104, **B**: *Sphingoterrabacterium* sp. SL-106, **C**: *Burkholderia* sp. SL-187, **D**: *Sphingobium yanoikuyae*, SL- 197, **E**: *Bacteriodetes* SL-WC2) isolated from a Scottish soil. Details about the isolation procedure, media etc. can be found in Langenheder et al. [21]. Bacterial strains were selected from a larger number of isolates according to the following criteria: (a) they could utilise glucose at comparable rates, (b) they were able to reduce tetrazolium violet but not inhibited by it, (c) they could be assigned to different phylogenetic groups and (d) they were insensitive to the washing procedure used for inoculum preparation. Triplicate communities were prepared in monoculture and in all possible combinations of species. Communities were assembled in 96-well microtitre plates at a final concentration of 2 × 10^8^ cells/ml on mineral salts medium containing 7.5 mM glucose as the only carbon source. Such high cell concentrations are close to the carrying capacity of the medium and were chosen to minimize the potential for strong growth effects during the experiment. Mixed communities were assembled using equal abundances of all component strains. All five strains could grow with glucose as sole carbon source and the medium was a mineral salts medium of Brunner (composition #457, http://www.dsmz.de/microorganisms/media-list.php), which was supplemented with 0.01% of the redox indicator tetrazolium violet (Sigma-Aldrich, St. Louis, USA). Inocula were prepared by growing strains from glycerol stock cultures and were then concentrated by centrifugation for 10 minutes at 12,800 × g. Cells were washed by re-suspension of pellets in mineral salts medium and further diluted to the required cell concentration using previously established calibration curves between optical density measurements and cell numbers as determined by DAPI staining and epifluorescence microscopy for each strain. Following establishment of all communities, half of the total volume (150 μl) of culture in each well was transferred to 96-well PCR plates, carefully sealed and placed into PCR Express thermal cyclers (Hybaid, Middlesex, UK) that were used as incubators. Communities were exposed to either a constant temperature of 22°C, or a temperature regime with one of two levels (± 4˚ or ± 8°C) of sinusoidal fluctuation around a mean of 22°C with a cycle interval of either 1 h or 5 h. Irrespective of temperature treatment, all plates were initially incubated at 22°C for 2 h. The total duration of the experiment was 42 h, which was chosen based on preliminary tests that showed that substrate depletion did not occur during this time period.

Respiratory activity was estimated at the end of the experiment by spectrophotometric (λ=600 nm) measurement of colour development, resulting from the reduction of tetrazolium violet, using a Thermomax microtitre plate reader (Molecular Devices, Wokingham, UK). Absorbance values were corrected for initial values measured directly after inoculation prior to statistical analysis. Prior to the experiment, we also performed tests to determine activity and growth of monocultures of the five strains over the temperature range used in the experiment.

### Statistical analysis

Four statistical models with respiratory activity as the dependent variable were developed. The first two models were based on data resulting from the constant temperature treatment, where the first model included species richness (SR, coded as 5 levels ranging from 1 to 5 species) and the second model included species composition (SC, coded as 31 distinct levels of all possible combinations of strains A, B, C, D and E) as the independent variable. The third model included species richness, the frequency of temperature change (F, coded as two levels, 1 hour and 5 hours) and the amplitude with which the temperature fluctuated around the mean (A, coded as two levels, ± 4˚ and ± 8°C), and the fourth model species composition, frequency of temperature change and amplitude of temperature change. The constant and variable temperature data could not be included in the same statistical framework as the constant temperature data has no degrees of freedom associated with frequency and amplitude of temperature variation. We are aware of approaches to include species richness and composition into the same model when, as is the case here, information about the performance of individual species in mixtures is missing [22,23]. However, these techniques require that either that the data complies to the assumptions of ANOVA [23] or that it follows a particular experimental protocol (random partitions design, Bell et al. 2009), both of which are not applicable to our study. Hence, the effects of species richness and species composition were tested in two separate models. Details of the model structure can be found in Additional file 1. Following initial linear regression models, Q-Q plots indicated normality of residuals, but plots of residuals verses fitted values revealed heterogeneity of variance. We therefore adopted the statistical approach of linear regression with a generalized least-squares (GLS) extension [24-26], which allows heteroscedastic variances (unequal variances among treatment combinations) to be modelled as a variance covariance matrix [25,26]. Following West et al. [26] and Zuur et al. [24], the most appropriate variance covariate matrix was determined using AIC scores in conjunction with plots of fitted values versus residuals with different variance covariate terms relating to the independent variables, using restricted maximum likelihood (REML, [26]). Once the appropriate random component had been determined, the fixed component of the model was refined by manual backwards stepwise selection using maximum likelihood (ML) to remove insignificant independent variable terms. The minimal adequate model was presented using REML [26]. Following Underwood [27], the highest order significant interactions in the minimal adequate model were examined, but nested levels within these were not examined. However, the importance of individual independent variables within each model was estimated using a likelihood ratio (L-ratio) test to compare the full minimal adequate model with a model in which the relevant independent variable, and all the interaction terms that it was involved in, was omitted. Analyses were performed using the ‘R’ statistical and programming environment and the ‘nlme’ package [28]. In order to assess whether there were any positive effects of species interactions (complementarity effects) on respiratory activity, we compared respiratory activity in species mixtures relative to the activity of the best performing monocultures using the overyielding metric D_max_[29]. D_max_ will be > 0 when a mixture performs better than the best performing component strain in monoculture. Activities of monocultures in various temperature fluctuation treatments were compared using 1-way ANOVA using the temperature treatments as grouping variables.

## Results

### (a) Activities of monocultures under constant temperature conditions

Strains A, D and E had highest activity at 26°C, strain B was most active at 22°C and strain C did not differ significantly over the temperature range studied (Additional file 2). All strains showed some activity over the entire temperature range, even though two of them (B and D) showed a rapid decline in activity when the temperature was increased from 26 to 30°C (Additional file 2).

### (b) Effects of species richness on respiratory activity

**Figure 1:**
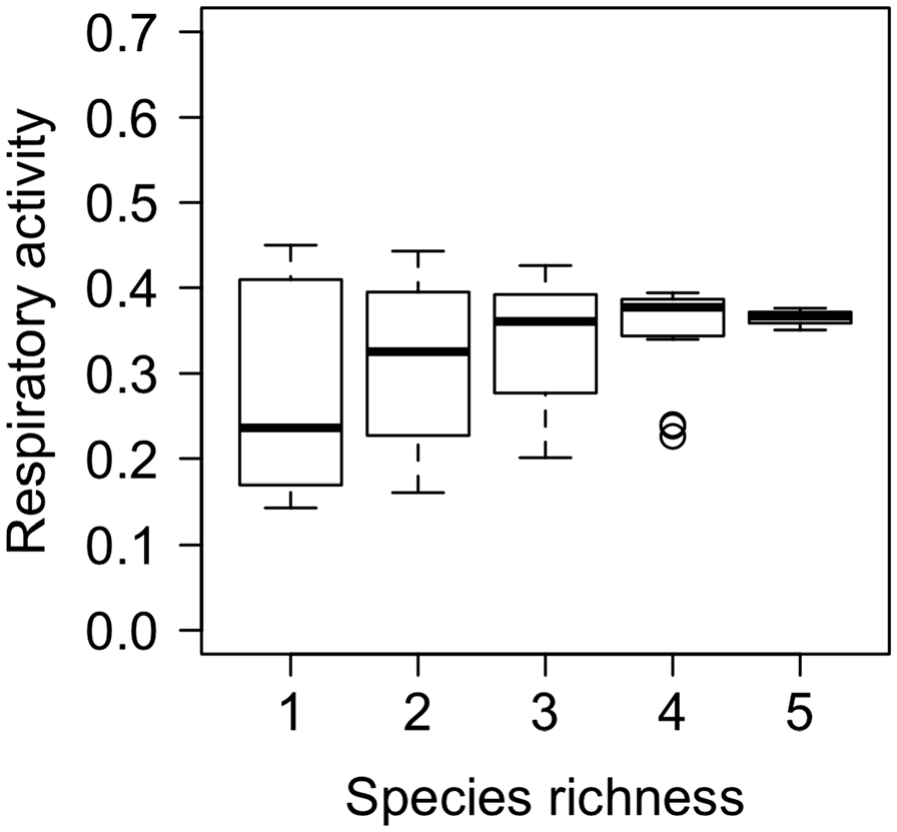
**Boxplots of the effects of species richness on respiratory activity under constant temperature conditions**.

Under constant temperature conditions, species richness had an overall positive effect on the mean respiratory activity, but successive levels of species richness were not always significantly greater than the previous level (Table 1, Figure 1).

**Table 1:** Summary of the statistical regression models used to investigate effects of species richness and species composition on respiratory activity under (A) constant and (B) varying temperature regimes

**A Constant temperature regime**				
*Independent variables*	*Significant terms*	*L-ratio*	*d.f.*	*p-value*
**1. SR**	SR	12.7	6	0.013
**2. SC**	SC	36	32	<0.0001
**B Varying temperature regimes**				
*Independent variable*	*Significant terms*	*L-ratio*	*d.f.*	*p-value*
**1. SR, A, F**	SR × A	30.1	18	<0.0001
	A × F	5.66	21	0.017
Importance of independent variables:				
SR		94.4	14	<0.0001
F		8.34	20	0.0154
A		37.8	16	<0.0001
**2. SC, A, F**	SC × A × F	132	156	<0.0001
Importance of independent variables:				
SC		1485	66	<0.0001
F		338	124	<0.0001
A		540	124	<0.0001

For each model, the highest order terms are presented and, where multiple terms are present, the relative importance of individual terms (and their interactions) within a model can be compared using the likelihood ratios (L-ratios). Note that separate models were run to test the effects of species richness (SR) and species composition (SC). A: amplitude of temperature change, F: frequency of temperature fluctuation. d.f.: degrees of freedom.

**Figure 2:**
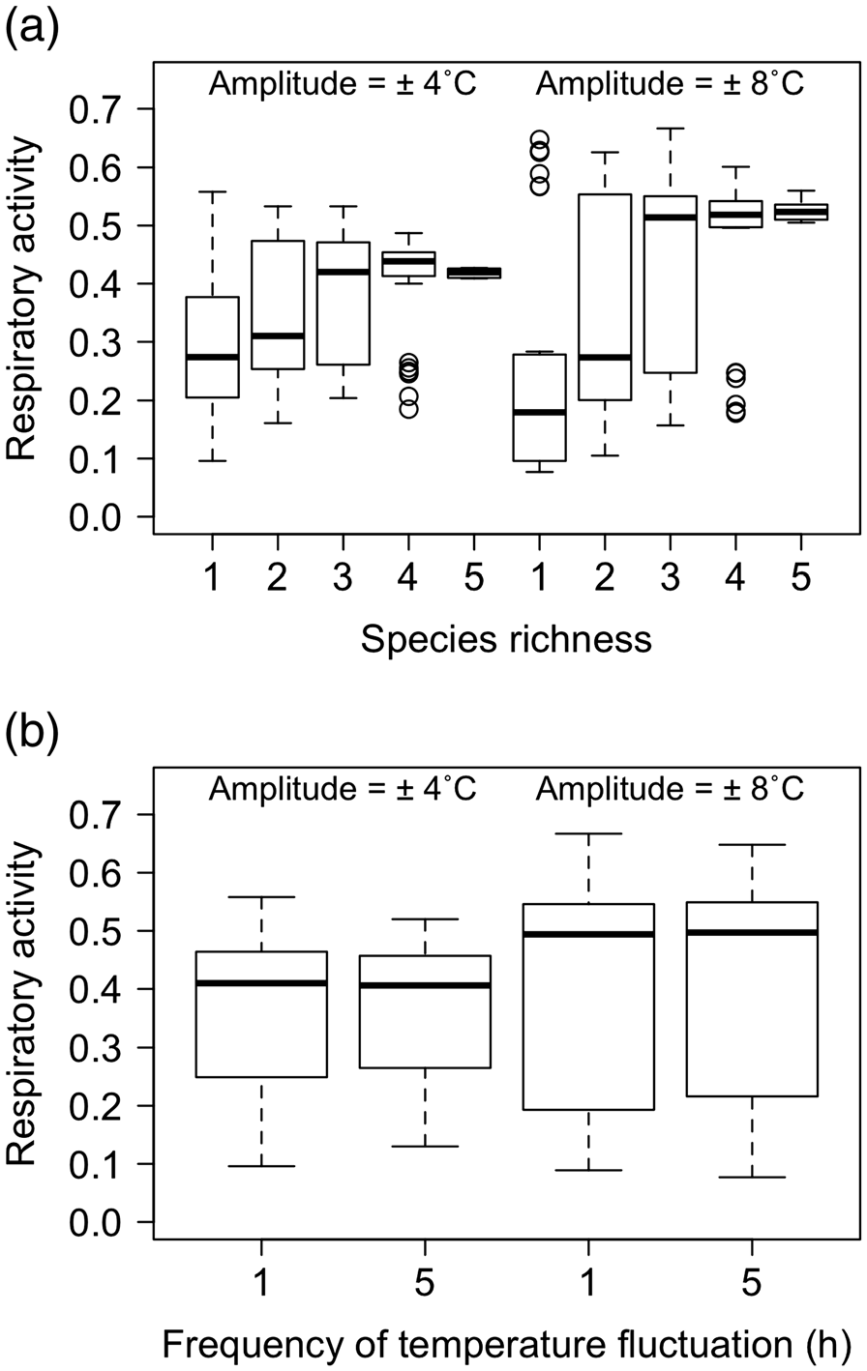
**Boxplots of the effects of species richness, frequency of temperature fluctuation and amplitude of temperature change on respiratory activity under varying temperature conditions.** The amplitude of temperature change (8: ± 4°C or 16: ± 8°C) is indicated within each panel.

Under varying temperature conditions, species richness had the greatest influence on respiratory activity, followed by the amplitude of temperature change and the frequency of temperature fluctuation, but these effects were dependent on two 2-way interactions (Table 1). We found a weak but significant effect of frequency of temperature fluctuation × amplitude of temperature change on respiratory activity (Table 1, Figure 2a) and strong effects of species richness × amplitude of temperature change (Table 1, Figure 2b). For the latter, the effects of species richness were positive but the form of the response was dependent on the amplitude of temperature change (Figure 2a), with respiratory activity increasing almost linearly with species richness at the higher amplitude but showing a saturating response at the lower amplitude (Figure 2a). These effects were most pronounced at the highest level of species richness and, relative to the constant temperature regime (Figure 1), an increased amplitude of temperature change had a positive effect on respiratory activity at all species richness levels.

### (c) Effects of species composition on respiratory activity

**Figure 3:**
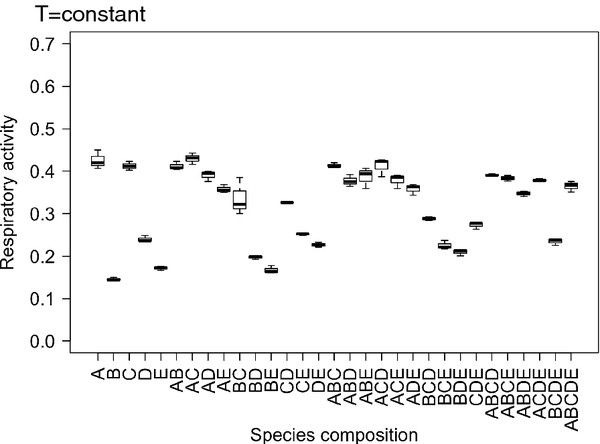
**Boxplots of the effects of species composition on respiratory activity under constant temperature conditions.** Species composition treatments are abbreviated following the coding outlined in Materials and Methods.

Under constant temperature conditions, differences in species composition had strong effects on respiratory activities (Table 1, Figure 3), where highest levels of activities were observed in communities that contained strain A and/or strain C (Figure 3).

**Figure 4:**
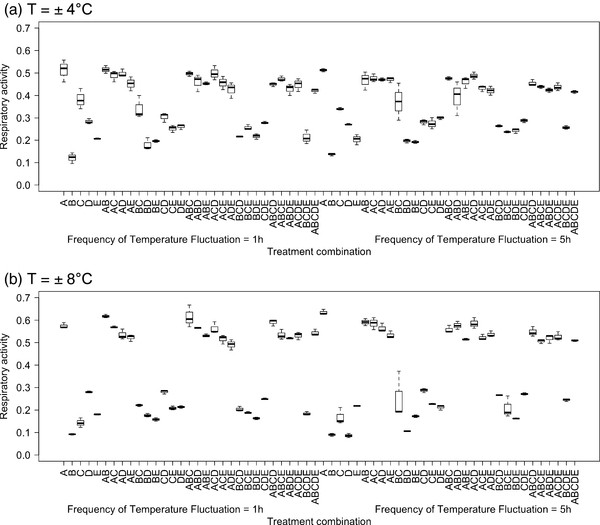
**Boxplots of the effects of species composition, frequency of temperature fluctuation and amplitude of temperature change on respiratory activity under varying temperature conditions.** Treatment levels on the x-axis are coded in the order species composition (species indicated by letters, A-E) and the frequency of temperature fluctuation (left = 1 h, right = 5 h). The effect of differences in the amplitude of temperature change can be evaluated by comparing panel (**a**) and (**b**), i.e. ± 4°C and ± 8°C amplitude respectively.

Also in case of the fluctuating temperature regime, the functional contributions that individual communities made varied greatly within each species richness level, and were influenced by both the amplitude of temperature change and the frequency of temperature fluctuation (Figure 4). By comparing the minimal adequate model (species composition × amplitude of temperature change × frequency of temperature fluctuation; Table 1) with models in which the components of temperature variability were excluded, we found that species composition was by far the most influential variable on respiratory activity, followed by the amplitude of temperature change and frequency of temperature fluctuation (Table 1).

**Figure 5:**
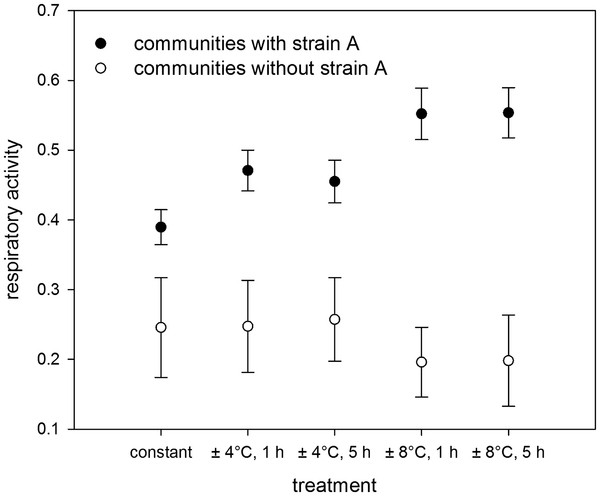
**Respiratory activities under different temperature regimes comparing communities with strain A and communities without strain A.** Differences were statistically significant (Wilcoxons rank sum tests, p < 0.0001) in all cases.

**Figure 6:**
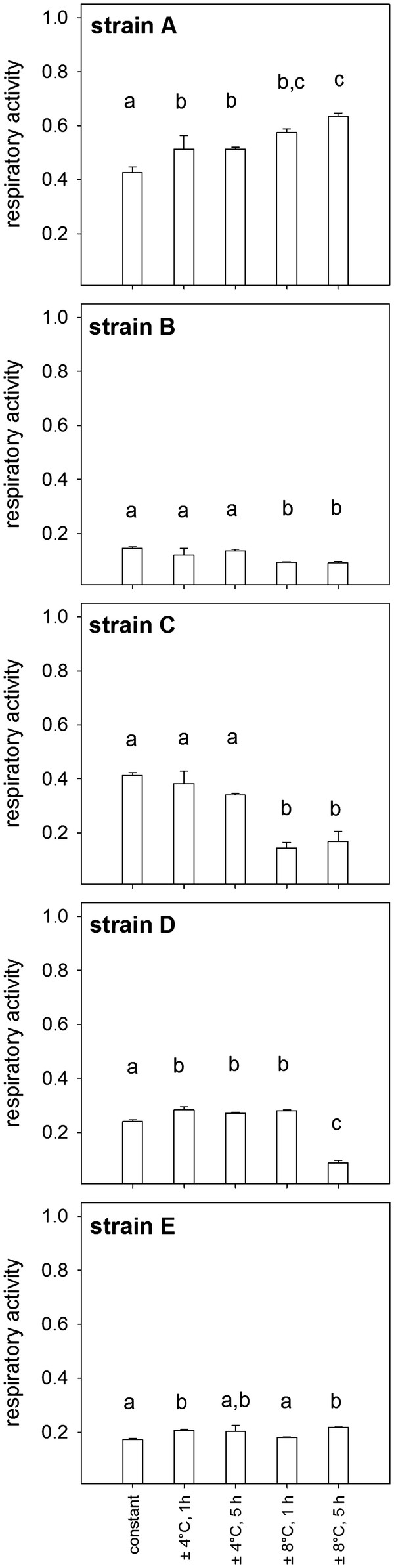
**Respiratory activity of monocultures under different temperature regimes.** Error bars indicate standard deviations calculated from 3 replicate cultures. Letters above bars indicate significantly different groups (*p* < 0.05) derived from a Tukey’s pairwise comparisons test following a 1-way ANOVA with the 5 different temperature fluctuation treatments as grouping variables.

At constant temperature, highest respiratory activity was observed in communities that contained strain A and/or strain C, whilst lowest metabolic activities tended to be observed when strain B was present. There were clear functional differences between communities that contained strain A compared to those that did not under constant temperature conditions and these differences were further exacerbated when the amplitude of temperature change was increased (Figure 5). The importance of strain A is also supported by inspection of the respiratory activity of the 5 strains in monoculture (Figure 6), which showed that there were overall differences in the metabolic activities of strains as well as clear differences in their response to varying temperature regimes. Strain A exhibited significantly higher activities in the high temperature fluctuation treatments (± 8°C), whereas activities of the other strains (strains B, C and D) tended to be lower or remained unchanged (strain E) compared to the control and low amplitude treatments (± 4°C). The effects of changes in the frequency of temperature fluctuation were inconsistent across communities that differed in species composition (Figure 4) and between different amplitude of temperature change treatments (compare Figures 4a and 4b).

Whilst respiratory activity in the majority (86%, n = 135) of the species mixtures showed no evidence of overyielding (D_max_ < 0), there were some cases, in particular in the high temperature fluctuation treatment (± 8°C) where communities overyielded (Additional file 3). There was no indication that overyiedling increased with increasing species richness.

## Discussion

Although the buffering and performance-enhancing mechanisms underpinning the insurance effects of biodiversity [4,5] are generally accepted as being equally plausible, experimental evidence for the latter is scarce. Here we performed an experiment to test how bacterial biodiversity-respiration relationships were influenced by the amplitude and frequency of a temperature change. In congruence with our hypotheses, we found that environmental variability strengthened the effect of species richness on ecosystem functioning and therefore provided clear evidence of a performing-enhancing role of biodiversity under fluctuating conditions.

In general, complementarity and selection effects can explain positive effects of species richness on ecosystem functioning [29,30]. Previous studies that have found an increase in ecosystem functioning under fluctuating environmental conditions due to niche complementarity and positive interactions [15,16], indicating several mechanisms might simultaneously operate and be of importance. Our results are, on the contrary, more consistent with the findings of Steiner et al. [31] who could show that species richness increased resilience of total community biomass after a perturbation due to rapid growth of a few dominant species. In order to be able to clearly separate selection and complementarity effects, information on the performance of individual species within a mixed community is required, which we lack here [29,30]. Nevertheless, our study does suggest ecosystem functioning increases under varying environmental regimes due to stronger selection effects, i.e. the increased relative performance of individual species. There was a marked increase in levels of ecosystem functioning with increasing amplitude of temperature fluctuation, associated with combinations involving strain A (Figure 5). Combinations without strain A had, on the contrary, similar or lower levels of ecosystem functioning under fluctuating compared to constant temperature conditions. Moreover, the activities of strain A in monoculture increased both with increasing temperatures (Additional file 2) as well as with increasing amplitudes of temperature fluctuation (Figure 6). On the other hand, there was a clear lack of effect, or decline, in activities of monocultures of all the other strains (Figure 6) as well as of any combinations that did not contain strain A (Figure 5). Positive D_max_ values indicating overyielding and thus complementarity effects were generally sparse (Additional file 3). If they were found, it was primarily in treatments with the highest amplitudes of temperature fluctuation and primarily in communities without strain A, indicating that complementarity effects might be more important in communities that are not strongly dominated by a particular species. It is, however, important to note that the system in general had a high level of functional redundancy, with all species able to utilize glucose, which might have limited the potential for complementarity effects, such as niche differentiation and facilitation. Nevertheless, our findings suggest that selection effects were more important than complementarity effects and that environmental change may lead to a situation where (i) particular species, in this case strain A (Figures 5 and 6), are better suited to varying environments, or (ii) some species, in this case strains B, C and D (Figures 5 and 6), are negatively affected, resulting in a competitive release from species which are otherwise functionally equivalent (redundant). Both outcomes result in an increasing dominance of the best performing species and, hence, an increase in ecosystem functioning. This also implies that performance-enhancing effects of biodiversity are likely to be influenced by changes in species interactions imposed by environmental circumstance, not functional capacity alone. Consequently, the level of ecosystem functioning achieved will largely depend on the contribution of a particular subset of species and how they interact to specific biodiversity-environment futures [32], emphasizing the need to consider species-specific responses to particular components (alone and in combination) of environmental change [33,34].

The context dependency of species interactions may, at least in part, also explain why negative or no effects of fluctuating environmental conditions on ecosystem functioning are often reported (e.g. [10,17,18,35,36]); thus, while there are environmental combinations that select one species over another, as in our study, there may also be conditions that exert neutral or negative selective pressures on other species. The fact that few congruent results have been documented to date may reflect the use of simplified model communities, where the selection of species with particularly traits may bias the effects that are observed. We therefore speculate that the performance enhancing effect may be limited to situations where environmental change and disturbances lead to the increasing dominance of generalists. Since natural ecosystems are increasingly dominated by generalists [37], our results might point towards an important mechanism that has hitherto not received much attention. In general, emphasis needs to be placed on investigation of the effects of the environmental context and perturbations on natural communities, since this knowledge will be essential in attempts to predict the effects of future environmental conditions on the delivery and magnitude of ecosystem services (e.g. [34,38-40]).

It is important to consider how communities respond to changes in the frequency of environmental fluctuation as well as the magnitude of change. Disturbance frequency has been found to affect diversity alone [41] and in combination with intensity [42,43], and therefore has the potential to affect functional properties of ecosystems indirectly. Our results showed that the intensity of a disturbance, i.e. the amplitude of the temperature change, was more important in determining respiratory activity than the frequency (= rate) of that change. This was probably because species richness effects were buffered by varying responses of individual species to changes in temperature frequency, i.e. strains were either slightly positively or negatively affected by higher disturbance frequency, depending on the magnitude of the temperature change. The fact that we observed interactive effects between species composition and different properties of the environmental regimes imposed here, supports the idea that insurance effects are not necessarily mediated by bulk properties of environmental change, but by multiple subtle properties of environmental forcing that are not necessarily immediately obvious [44]. However, since the change of the temperature amplitude imposed here does not necessarily match temperature profiles typically observed in nature, future studies should investigate whether similar effects and underlying mechanisms exist and are of generic importance in naturally assembled communities.

It is important to consider the methodological limitations of our study within the context of ecological theory. Theory predicts that the performance enhancing effect of biodiversity will lead to an increase of the mean of an ecosystem function over time [4], yet our study considered a point measurement of ecosystem functioning at the end of the experiment rather than monitored temporal changes in ecosystem functioning throughout the duration of the experiment. Thus, whilst our design did not include multiple time points required to demonstrate performance enhancing effects as a result of negative covariances among species, our results do incorporate multiple generations and are consistent with what would be predicted when such effects are present.

Bacteria have very short generation times and can rapidly adjust their physiology in response to changes in environmental conditions. This also means, however, that it was necessary to set the initial bacterial abundance close to that of the carrying capacity of the different bacterial strains to avoid a batch culture situation; an initial period of rapid growth of the most competitive strain would have lead to strong changes in evenness. By using relatively high initial abundances we ensured that the shift in dominance that we observed resulted from more realistic changes in the densities of species (e.g. due to release of nutrients when cells were increasingly inhibited at higher temperature fluctuations) or were due to differences in the rates at which species contribute to ecosystem functioning. One limitation of our study is that we did not measure the realized diversity in the community at the end of the experiment, hence, strictly speaking our results refer to relationships between initial diversity and respiratory activities. However, it is unlikely that our findings reflect changes in diversity caused by expirations of individual strains given the short incubations time (42 hours), high initial abundances of all components strains, and that all strains showed activity across all treatments, including those with the highest level of temperature fluctuations (Figure 6). Another limitation is the low diversity compared to natural bacterial communities that greatly exceeds the richness levels that are possible to include in experiments with tractable model communities [45]. Thus, while we have used a model system to identify the plausibility of a specific mechanism [46], an important next step will be to test whether performance enhancing effects of diversity also operate in natural bacterial communities exposed to environmental change and perturbations.

Increasing temperature can decrease species richness of communities [47], induce changes in community composition and facilitate temporal species turnover [48] and decrease community evenness [49], but it is clear that there are multiple factors that, in concert with biodiversity, will determine how climate change affects the provision and stability of ecosystem functioning [34,38,50]. Our study also confirms that subtleties in individual factors, in particular the amplitude and frequency of the change, may affect community structure and, subsequently, ecosystem functioning [33]. This may result in an increasing dominance of particular key species that facilitate ecosystem functioning under certain alternative environmental conditions. Collectively, our findings emphasise the need to consider how species, alone and in combination, respond to and interact with, multiple properties (variance, extremes, cycles etc.) of a changing environment in order to reduce the uncertainty associated with predicting the functional consequences of biodiversity-environmental futures.

## Conclusions

Here we show that positive effects of species richness on ecosystem functioning are enhanced by environmental fluctuations due to stronger selection towards functionally dominant species. This finding indicates that functional over-compensation by dominant species might be an important mechanism maintaining ecosystem functioning under environmental change.

## Competing interests

The authors declare that they have no competing interests.

## Authors’ contributions

SL, MTB, JIP and MS designed the research, SL implemented the experimental work, SL, MTB and MS analysed the data, and SL and MS wrote the manuscript with input from MTB and JIP. All authors read and approved the final manuscript.

## Supplementary Material

Additional file 1 details of the structure of the models. Click here for file

Additional file 2 A figure showing respiratory activities of strains that were incubated for 42 hours at temperatures resembling those that were used in the main experiment. Click here for file

Additional file 3 **Predicted yields (D**_**max**_**) in respiratory activity of mixed communities relative to monocultures for constant and fluctuating temperature regimes.** Click here for file
