# Supplementary material for: Role of functionally dominant species in varying environmental regimes: evidence for the performance-enhancing effect of biodiversity
Source: BMC Ecol. 2012 Jul 30;12:14. doi: 10.1186/1472-6785-12-14 (PMC3480835; doi:10.1186/1472-6785-12-14)

Additional file 1

Figure showing the respiratory activities of strains that were incubated for 42 hours at temperatures resembling those that were used in the main experiment. Respiratory activity was determined, spectrophotometrically at 600 nm, as the colour change resulting from reduction of a redox dye added to the culture medium. Note that these cultures were incubated in different incubators so that results are not directly comparable to those from the main experiment. Error bars refer to standard deviations calculated from 3 replicate cultures.

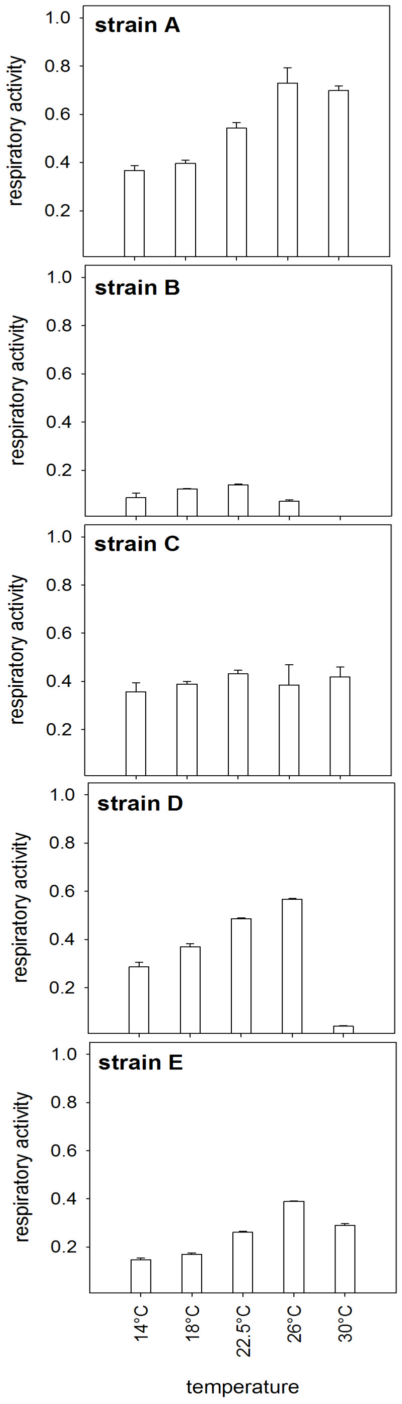

Supplement: Additional file 1 — details of the structure of the models. [file 1472-6785-12-14-S1.pdf]
