# Supplementary material for: Role of functionally dominant species in varying environmental regimes: evidence for the performance-enhancing effect of biodiversity
Source: BMC Ecol. 2012 Jul 30;12:14. doi: 10.1186/1472-6785-12-14 (PMC3480835; doi:10.1186/1472-6785-12-14)
Supplement: Additional file 2 — A figure showing respiratory activities of strains that were incubated for 42 hours at temperatures resembling those that were used in the main experiment. [file 1472-6785-12-14-S2.pdf]

Additional file 2. Details of the structure of the models

Model 1. Form of (a) the initial linear regression model and (b) the minimal adequate linear regression model with generalised least-squares (GLS) extension (incorporating species richness as a variance-covariate) for the effects of species richness (SR) on respiratory activity (RA) under constant temperature conditions.

(a) `RA ~ as.factor(SR)`

(b) `RA ~ as.factor(SR), weights = varIdent(form = ~ 1|as.factor(SR)), method = "REML")`

Model 2. Form of (a) the initial linear regression model and (b) the minimal adequate linear regression model with generalised least-squares (GLS) extension (incorporating species richness as a variance-covariate) for the effects of species composition (SC) on respiratory activity (RA) under constant temperature conditions.

(a) `RA ~ as.factor(SC)`

(b) `RA ~ as.factor(SC), weights = varIdent(form = ~ 1|as.factor(SC)), method = "REML")`

Model 3. Form of (a) the initial linear regression model and (b) the minimal adequate linear regression model with generalised least-squares (GLS) extension (incorporating species richness and temperature fluctuation as variance-covariates) for the effects of species richness (SR), frequency of temperature change (F) and amplitude of temperature change (A) on respiratory activity (RA) under varying temperature conditions.

(a)
```
RA ~ as.factor(SR) + as.factor(F) + as.factor(A) +
as.factor(SR):as.factor(F) +
as.factor(SR):as.factor(A) +
as.factor(F):as.factor(A) +
as.factor(SR):as.factor(F):as.factor(A)
```

(b)
```
RA ~ as.factor(SR) + as.factor(F) + as.factor(A) +
as.factor(SR):as.factor(A) +
as.factor(F):as.factor(A),
weights = varIdent(form = ~ 1|as.factor(SR) * as.factor(A)), method = "REML")
```

Model 4. Form of (a) the initial linear regression model and (b) the minimal adequate linear regression model with generalised least-squares (GLS) extension (incorporating species composition and temperature fluctuation as variance-covariates) for the effects of species composition (SC), frequency of temperature change (F) and amplitude of temperature change (A) on respiratory activity (RA) under varying temperature conditions.

(a)
```
RA ~ as.factor(SC) + as.factor(F) + as.factor(A) +
as.factor(SC):as.factor(F) +
as.factor(SC):as.factor(A) +
as.factor(F):as.factor(A) +
as.factor(SC):as.factor(F):as.factor(A)
```

(b)
```
RA ~ as.factor(SC) + as.factor(F) + as.factor(A) +
as.factor(SC):as.factor(F) +
as.factor(SC):as.factor(A) +
as.factor(F):as.factor(A) +
as.factor(SC):as.factor(F):as.factor(A),
weights = varIdent(form = ~ 1|as.factor(SC) * as.factor(A)), method = "REML")
```
