# Supplementary material for: Role of functionally dominant species in varying environmental regimes: evidence for the performance-enhancing effect of biodiversity
Source: BMC Ecol. 2012 Jul 30;12:14. doi: 10.1186/1472-6785-12-14 (PMC3480835; doi:10.1186/1472-6785-12-14)
Supplement: Additional file 3 — Predicted yields (Dmax) in respiratory activity of mixed communities relative to monocultures for constant and fluctuating temperature regimes. [file 1472-6785-12-14-S3.pdf]

Additional file 3

Figure showing predicted yields (D_{max}) in respiratory activity of mixed communities relative to monocultures for environmental regimes where temperature is (a) at constant temperature of 22˚C, or tracks a sinusoidal fluctuation around a mean of 22˚C of (b) ±4˚ with a cycle interval of 1 h, (c) ±8˚C with a cycle interval of 1 h, (d) ±4˚ with a cycle interval of 5 h or (e) ±8˚C with a cycle interval of 5 h. The vertical lines indicate D_{max} = 0. Confidence intervals (95%) for D_{max} were estimated using a variation of the second percentile bootrapping method using the model prediction for metabolic activities as we have done earlier [1]. In the first step, the order of observed model residuals for the metabolic activity models was randomised and then added to the original model estimates. The procedure of deriving a linear regression with a GLS extension was then applied to these data and the whole process was repeated 1000 times. Within each of those 1000 iterations, D_{max} values were calculated, and the distribution of bootstrapped D_{max} values were then used to estimate confidence intervals for the original D_{max} estimates.

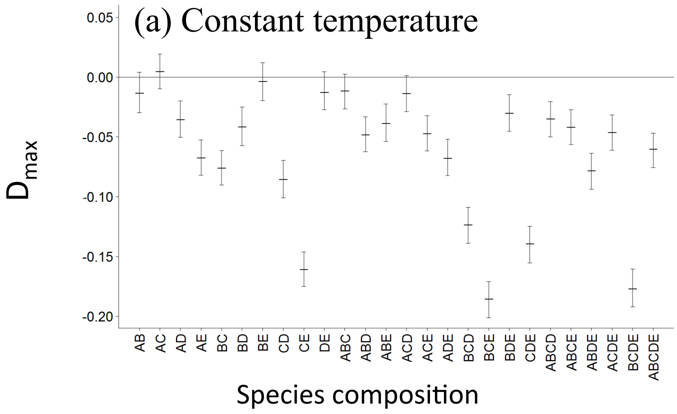

(b) – (e) Fluctuating temperature

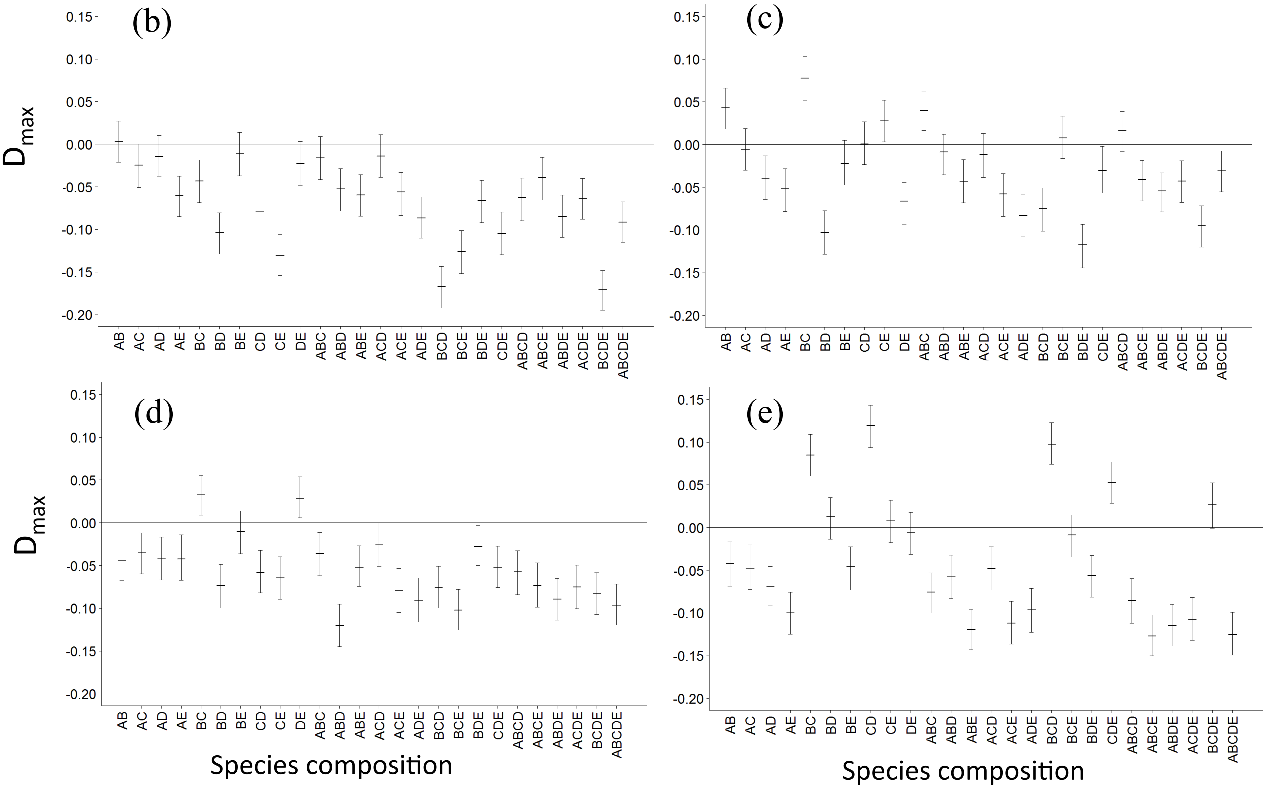

Reference

1. Langenheder S, Bulling MT, Solan M, Prosser JI: **Bacterial Biodiversity-Ecosystem Functioning Relations Are Modified by Environmental Complexity**. *Plos One* 2010, **5**:e10834.
